# Social Medicine in the Twenty-First Century

**DOI:** 10.1371/journal.pmed.0030445

**Published:** 2006-10-24

**Authors:** Scott Stonington, Seth M Holmes, The PLoS Medicine Editors

## Abstract

The*PLoS Medicine* editors, and Guest Editors Scott Stonington and Seth Holmes, introduce a special collection of articles examining the social structures that contribute to illness and health.

In its launch issue in October 2004, *PLoS Medicine* signaled a strong interest in creating a journal that went beyond a biological view of health to incorporate socioeconomic, ethical, and cultural dimensions. For example, that first issue contained a policy paper on how the health community should respond to violent political conflict [1], a debate on whether health workers should screen all women for domestic violence [2], and a study on the global distribution of risk factors for disease [3].

Two years on, our October 2006 issue takes our interest even further. It contains a special collection of ten magazine articles and five research papers devoted entirely to social medicine. We are delighted that the collection features many of the leaders in the field, including the renowned medical anthropologists Paul Farmer and Arthur Kleinman, the former United States Surgeon General David Satcher, and the Harvard professor of social medicine and psychiatry Leon Eisenberg.

Most of our readers have welcomed our inclusive view of what a medical journal should highlight. Some, however, have been critical, suggesting that we should publish “less soft stuff” and more “hard science.” These critics might argue that in this era of stem cell research and the human genome project, of molecular medicine and DNA microarray technology, the notion of social medicine seems irrelevant and outmoded.

But the ultimate role of a medical journal is surely to contribute to health improvement, and that means looking not just at molecules but at the social structures that contribute to illness. The stark fact is that most disease on the planet is attributable to the social conditions in which people live and work [4]. The socially disadvantaged have less access to health services, and get sicker and die earlier than the privileged. Despite impressive technological advances in medicine, global health inequalities are worsening.

“All medicine is inescapably social,” said Leon Eisenberg [5], and we entirely agree. Take, for example, the announcement of the sequencing of the human genome, which the BBC predicted would mean we could “banish inherited disorders, screen people for their vulnerability to diseases, tailor treatment to an individual's genetic make-up, create thousands of new drugs and extend human lifespan” [6]. In time, perhaps these predictions will be partly or fully realized. What is certain, though, is that the human genome project has also opened up an immediate Pandora's Box of complex ethical, legal, and social issues. These issues include ensuring equity in patients' access to the fruits of the project and balancing the benefits, risks, and economic costs of genetic screening. Even the human genome is “inescapably social.”

And so, to complement the papers on molecular medicine that we have published over the last two years—such as papers on genetic mutations that confer resistance to cancer drugs [7,8] or on differentiation of insulin-producing cells from human neural progenitor cells [9]—we have also dedicated space in the journal to considering the large-scale social forces that give rise to human disease and affect its distribution around the globe. These include economics, politics, legal institutions, and power structures.

Throughout our special collection, one pioneer in understanding these large-scale social forces is repeatedly acknowledged—Rudolf Virchow. In his 1848 medical report of an outbreak of typhus in Silesia, Virchow concluded that poverty and living conditions, not biology, were the prime causes of the epidemic [10]. While an understanding of these large-scale forces remains social medicine's base and one of its most important tasks, this special collection shows the ways in which finer-grained social forces have an equally important effect on health. The different levels at which social forces operate can be considered as four primary domains, beginning in the clinical encounter and opening outward to society and the globe.

The first domain, then, is made up of the cultural and social aspects of the relationship between patients and health professionals. This relationship is a social negotiation affected by beliefs, practices, interests, and power dynamics. Communication within this relationship can have a powerful impact upon health outcomes. The influence of this relationship upon health is not limited to Western, allopathic, biomedical systems but is equally as important in other medical systems throughout the world [11].

The second domain involves patients' beliefs, practices, and experiences. Patients' experiences of and responses to suffering are not confined to the clinical encounter and vary dramatically among different populations. Understanding the specifics of people's everyday lives is essential to engaging with them and their illnesses [12,13].

The third domain is the culture of medicine itself. Health professionals and institutions have their own cultures that also go beyond clinical interactions. Health systems and health research both contain agendas, prejudices, and beliefs that can lead to certain perspectives being favored as the most legitimate. Understanding the culture of medicine is essential to understanding health professionals' attitudes toward illness, patients, and treatments [14–16].

The final domain brings us, full circle, back to Virchow and the large-scale forces that shape health, which have become known as the social determinants of disease. The 15 papers in the special collection each relate to at least one of these four domains, as shown in Table 1.

**Table 1:**
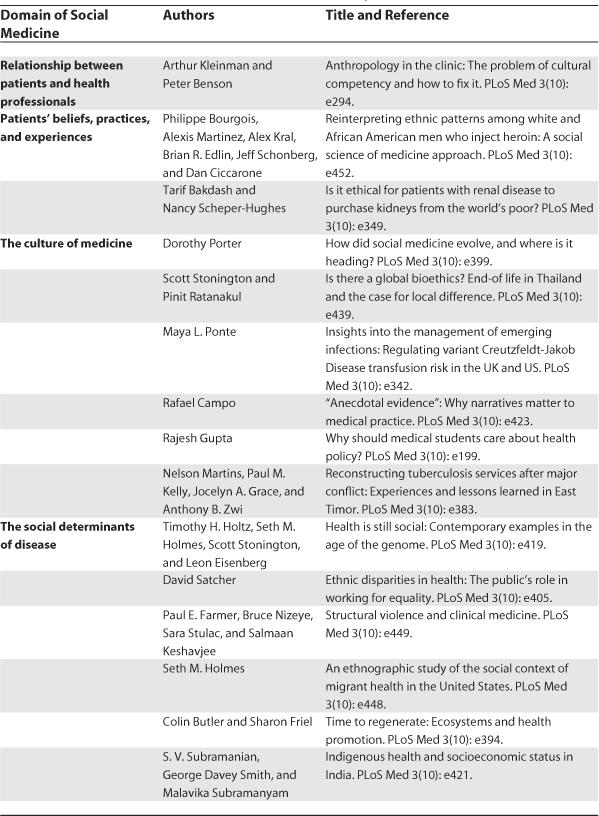
The Four Domains of Social Medicine in the Special Collection

In their compelling policy paper for this special collection, Paul Farmer and colleagues argue that while the search for the molecular basis of disease has been enormously fruitful, there has at the same time been a “desocialization” of scientific inquiry. In other words, they say, there has been “a tendency to ask only biological questions about what are in fact *biosocial* phenomena” [17]. We hope that our special collection helps to “resocialize” our understanding of disease distributions and outcomes.
